# Case Illustrating the Influence of the Imagination upon the Voluntary Muscles

**Published:** 1877-04

**Authors:** W. H. DeWitt

**Affiliations:** Physician at Longview Asylum


CASE ILLUSTRATING THE INFLUENCE OF THE IMAGINATION UPON THE VOLUNTARY MUSCLES.—*By W. H. De Witt, M.D., Physician at Longview Asylum.*—Elizabeth C., native of Ohio, aged 27 years, single, occupation, none.

The following history was obtained from the patient and her relatives: A maternal uncle and an aunt are insane; a brother died of epilepsy at the age of 35. The patient has been an invalid for five years; has only occasionally been out of bed, and during the five years has not walked a step or borne a pound of weight upon her limbs.

She is a woman of ordinary height, poorly nourished, anæmic, tissues soft and flabby; light complexion, light hair, and blue eyes; upon the whole, rather attractive in appearance; no preternatural heat of the body, pulse frequent and irritable, respirations 22 per minute, tongue frosted, bowels constipated.

The theme of her conversation is upon matters relating to herself and religion. She converses in a most positive, audible tone of voice. Her delusions are of the following character: She firmly believes that she is influenced and governed by some supernatural power, and that her actions are created and controlled by this power. She also imagines that she is suffering from a great variety of physical disorders, such as spinal disease, uterine trouble, etc.; in fact, there is scarcely a disease in the medical vocabulary from which she has not suffered. She affirms that she is not to walk until a higher power than man commands it. Her afflictions have been placed upon her for a wise purpose, to chasten her, and when their mission is fulfilled she will astonish the whole civilized world by her marvelous works. She is a most firm believer in miracles, and affirms one will be wrought upon her.

A most careful examination of her body revealed nothing unusual or of a special abnormal character; absence of both spinal or uterine disease. The muscles of the body have become somewhat atrophied and weakened, more especially those of the lower limbs, no doubt the result of disease. She has not, however, lost the use of any portions of the body; changes her position in bed without assistance, uses her arms and legs with perfect freedom. There seems to be no muscular inhibition worth speaking of, but simply a lack of force or vigor, brought about by want of exercise. The interrupted or faradaic current applied to the extremities produced electro-muscular contraction.

Believing that the inability to walk was in a great measure imaginary, and feeling fully satisfied that there were no physical causes operating to prevent the use of her limbs, I resolved to resort to some stratagem, knowing that her credulity would permit of any amount of imposition being practiced. As before stated, she had long been impressed with the belief that her physical ailments were to be removed by divine interposition, or in her words, the Almighty would restore her to perfect physical health, and at the same time *command* her to walk. Acting upon the supposition that she was led to believe that it had been revealed in a dream that on the night of the 23d of the eighth month (August) the manifestations of divine power she had so long expected would be made; in pursuance of the above all the necessary preparations were made to have a first-class miracle. A tube of considerable length was procured, also materials for lighting up her room. Her faith seemed strong in the truth of the dream, and she awaited the 23d with visible anticipation. The proper time having arrived, the tube was introduced through the window of her room, as nearly over the head of her bed as possible, and the following words enunciated in a solemn and impressive tone: "Elizabeth, daughter of Rebecca, the spirit speaketh unto thee. Thou hast suffered these many years. For my sake thou hast been patient, and now thy *faith* shall make thee whole. Thou hast a great and good work to accomplish; I therefore command thee to walk. From this time forth thou shalt be my servant." Withdrawing the tube, the room was at once illuminated in a most brilliant and dazzling manner. It need only be said that the stratagem worked well. She was found by the night watch walking about her room in a most happy frame of mind; was fully satisfied with the genuineness of the miracle, but believed that others would be performed at no distant time.

From this time forward, up to present date (January 19), she has been walking with the aid of a cane. Her general health has improved very much; her limbs have increased in size and grown comparatively quite strong. She is still at times exceedingly nervous and hysterical. Her mental condition has improved some; she still, however, has delusions, thinks that all has not been done for her that will be done, and is looking forward to other manifestations.

This case clearly illustrates the powerful influence of imagination over the voluntary muscles. The young woman labored under the belief for five years that she could not walk. The belief was created and perpetuated, no doubt, by the delusion that she could not walk until commanded to do so. There are two reasons given by Dr. Carpenter by which actions may become impossible that under ordinary circumstances are performed with ease. First, if a man's mind be entirely possessed with the idea of its *impossibility;* or secondly, if, while his judgment entertains no doubts of success, his attention be distracted by a variety of objects, so that he cannot bring it to bear upon the one effort which may alone be needed. In this particular instance, as in the first example, the mind was no doubt imbued with its impossibility, and therefore no effort was made to use the limbs as a means of support to the body. —*Cincinnati Lancet and Observer.*

